# High grade B-cell gastric lymphoma with complete pathologic remission after eradication of helicobacter pylori infection: Report of a case and review of the literature

**DOI:** 10.1186/1477-7819-6-35

**Published:** 2008-03-19

**Authors:** Luigi Cavanna, Raffaella Pagani, Pietro Seghini, Adriano Zangrandi, Carlo Paties

**Affiliations:** 1 Medical Oncology-Hematology Department, Hospital of Piacenza, 29100 Piacenza, Italy; 2 Department of Pathology, Hospital of Piacenza, 29100 Piacenza, Italy

## Abstract

**Background:**

Treatment of primary gastric diffuse large B-cell lymphoma is still controversial. The treatment of localized disease was based on surgery alone, or followed by chemotherapy and/or radiotherapy. High-grade gastric lymphomas are generally believed to be *Helicobacter pylori *(HP)-independent growing tumors. However a few cases of regression of high-grade gastric lymphomas after the cure of *Helicobacter pylori *infection had been described.

**Case presentation:**

We report here a case with primary diffuse large B-cell lymphoma that showed a complete pathologic remission after HP eradication and we reviewed the literature. A computerized literature reach through Medline, Cancerlit and Embase were performed, applying the words: high grade gastric lymphoma, or diffuse large B cell, MALT gastric lymphoma, DLBCLL (MALT) lymphoma and Helicobacter. Articles and abstracts were also identified by back-referencing from original and relevant papers. Selected for the present review were papers published in English before January 2007.

**Conclusion:**

Forty two cases of primary high grade gastric lymphoma that regressed with anti HP treatment were found. There were anedoctal cases reported and patients belonging to prospective studies; four trials studied the effect of eradication of *Helicobacter pylori* as first line therapy in high grade gastric lymphoma: 22 of a total of 38 enrolled patients obtained complete remission. Depth of gastric wall infiltration and clinical stage were important factors to predict the response to antibiotic therapy. Our case and the review of the literature show that high-grade transformation is not necessarily associated with the loss HP dependence. In early stage, for high-grade B-cell HP-positive gastric lymphomas, given the limited toxicity of anti-HP therapy, this treatment may be considered as one of the first line treatment options.

## Background

*Helicobacter pylori *(HP) infection plays an important role in the development and growth of gastric mucosa-associated lymphoid tissue (MALT) lymphomas [1,2]. Eradication of HP infection has been shown to result in durable tumor regression in 77% of patients with low-grade gastric MALT lymphoma [3].

It has been demonstrated by laboratory and clinical studies that primary gastric large B cell MALT lymphomas are transformed, antigen independent, autonomously growing tumors that are unlikely to respond to eradication therapy of the HP infection. An *in vitro *study by Hussell *et al *[4] showed that tumor cells from high grade gastric lymphoma did not respond to a co-stimulation of autologous T cells and lysate of a specific HP strain, as low grade gastric MALT lymphoma cells did. In addition, these results are also supported by the finding that most cases of antibiotics-resistant low grade MALT lymphoma contained an high grade component in the deeper layer of the gastric wall in their gastrectomy specimen [5,6].

However anedoctal cases of primary gastric large B-cell lymphoma that responded to antibiotic therapy had been described and, more recently, Chen *et al *[7] reported in a prospective study the disappearance of primary gastric large B-cell lymphoma at gastroscopy examination in 14 of 22 patients (64%) after HP eradication therapy.

We report here a patient with diffuse large B cell lymphoma of the stomach, that achieved a complete pathologic remission after anti HP therapy and a detailed review of literature is also presented.

## Case presentation

In May 2003, a 43-year-old man was admitted for epigastric pain of two months duration and weight loss (more than 10% of the body weight). Clinical examination was unremarkable and laboratory data were within normal values; only a mild hypochromic anemia was disclosed (Hb 12.4 g/dl).

**Figure 1:**
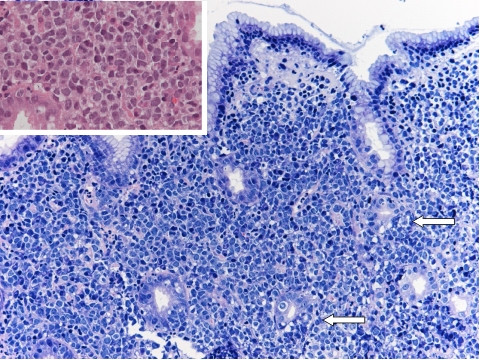
Histology before triple therapy shows antral gastric mucosa exhibiting interstitial infiltrate composed of large sized centroblast-like lymphoid cells (*inset*), with occasional lymphoepithelial lesions (*arrows*) (Giemsa, inset H&E ×200).

A gastroscopy was performed and revealed an ulcerative lesion in the gastric antrum ranging 3 cm in diameter. Biopsies established the diagnosis of diffuse large B cell lymphoma (DLBCL) of the stomach and *Helicobacter pylori *was identified in the mucosa. The previously reported diagnostic criteria for gastric diffuse large B-cell lymphoma were used [7,8] (Figure 1).

Endoscopic ultrasonography (EUS) showed a hemicircumferencial thickness of the anterior gastric wall, which was infiltrated until to the serosa. Staging was completed with neck, chest and abdominal computed tomography and with bone marrow biopsy. There were not other lymphoma-deposits outside the stomach, and a clinical stage E I_2 _was established.

The patient refused chemotherapy and a surgical treatment was then planned. Waiting this treatment, the patient underwent an HP eradication therapy. He received a triple therapy with omeprazole (20 mg twice a day), amoxicillin (1 g twice a day) and clarithromycin (500 mg twice a day) for seven days, and after that omeprazole (20 mg every day) for other 21 days.

**Figure 2:**
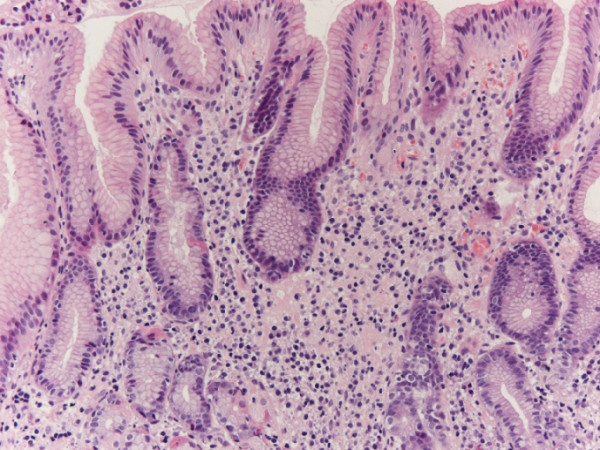
Histology after triple therapy shows antral gastric mucosa with sparse lymphoplasmacellular interstitial infiltrate, without evidence of lymphomatous cells (H&E ×200).

Prior to surgery, the patient underwent repeat gastroscopy (a month later) that showed a clear improvement of the ulcerative lesion of the gastric antrum and biopsies showed a complete disappearance of the lymphoma (Figure 2).

The patient was informed of the good results from anti HP therapy but he preferred to undergo to subtotal gastric resection. The histological examination revealed complete remission of the lymphoma and absence of Helicobacter pylori. He did not receive additional treatment and is in continuous complete remission after 42 months.

## Review of literature

We selected all cases reported with primary gastric large B-cell lymphoma treated with anti HP treatment and all cases of primary gastric large B-cell lymphoma treated in prospective studies with anti HP-therapy. According to the WHO classification, low grade MALT lymphoma with focal high grade component constituted by "solid or sheet-like proliferations of transformed cells" were included as diffuse large B-cell lymphoma [8].

Tumors were staged clinically according to the modified by Musshoff  and  Schmidt-Vollmer, Ann Arbor Classification [9] for extranodal lymphomas. Response rate were analyzed only if patients were included in prospective studies.

## Results

A total of 61 patients, including the present case, with primary gastric large B-cell lymphoma were treated with anti HP treatment [7,10-25] and 42 of them showed a complete response. There were anecdotal cases reported and patients belonging to prospective studies. Four trials studied the effect of eradication of *Helicobacter pylori *as first line therapy in gastric high grade gastric lymphoma: 22 of a total of 38 (57.9%) enrolled patients obtained complete remission. Data of the 42 responsive patients are reported in Table 1.

**Table 1:** Clinico-pathologic characteristics of 42 patients with high-grade B-cell gastric lymphoma responsive to eradication therapy

Age, median range year	59 (21–85)
Sex, Male/female	20/20, 2 not reported
Location of tumor (s), n (%)	
Antrum	15 (35.71)
Middle and/or lower body	17 (40.47)
More than two components	10 (23.80)
stage	30 (71.4) EI_1_
	6 (14.3) EI_2_
	3 (7.1) EII_1_
	1 (2.4) EIII
	2 not reported
Deaph of gastric wall involvement n(%)	
Submucose or above	21 (50)
Muscolaris propria or beyond	12 (28.57)
	9 not reported

Different schedules of eradication treatment were used and were based on a proton pump inhibitor (omeprazole, lansoprazole, or rabeprazole) together with a combination of antibiotics (clarithromycin, amoxicillin, and/or metronidazole). Forty-two of 61 patients obtained a complete remission of the lymphoma. In two patients there was gastric complete remission (despite of persistence of *Helicobacter pylori *in one patient) with remaining nodal disease. In one patient, large B cells disappeared, but areas of MALT lymphoma and nodal disease persisted. The patient with Burkitt-like lymphoma, obtained a complete remission.

Two patients were affected by AIDS [15,18]. In one of these patients, the eradication treatment was started together with antiretroviral therapy (stavudine, lamivudine and indinavir). Both patients obtained, almost initially, a complete remission.

The median time to remission of lymphoma, calculated on data available from 31 patients, was 8 weeks from the end of the eradication treatment. The median time to complete response reported by Chen *et al., *[7] was 9.6 months (range 0.0 to 20.4) for DLBCL (MALT) with low-grade predominant and 5.5 months for DLBCL (MALT) predominant.

Initial or complete regression of lymphoma was evident at the first gastroscopic examination (in most cases 4–8 weeks after the end of eradication treatment) in the majority of patients; only in one patient, there was a progression of disease after an initial partial response [22].

Four patients including present case underwent subtotal or total gastrectomy, after endoscopic confirmation that Helicobacter pylori infection was cured and lymphoma regressed [15,22].

Other patients in complete remission didn't undergo further treatment, except one patient with AIDS who relapsed after 6 months and needed chemotherapy. This is the only one relapse described. Two patients, in partial remission after eradication treatment, gained complete regression of lymphoma after chemotherapy [15,22]. Because of the advanced age, additional chemotherapy was postponed in a patient; "wait and watch" follow-up was chosen for him [15].

The median period of follow-up was 22 months. The longer period of follow-up was reported in the series of Chen *et al*., [7]: all the 14 DLBCL (MALT) patients with CR remained relapse-free after a median follow-up of 63 months.

Information about genetics of large B cells didn't express bcl-6 and p53; in the patient with Burkitt-like lymphoma, malignant cells expressed bcl-6 and p53; in the with Burkitt-like lymphoma, malignant cells expressed bcl-6 and not bcl-2; in two patients there were not alterations of p53 and k-ras genes and microsatellite instability [16].

In 20 patients, tumor response was unexpected, but in 22 cases it was obtained in prospective trials. Chen *et al*., [7] reported 14 cases, Nakamura *et al*., [10] 5 cases, Hiyama *et al*., [16] 2 cases and Alpen *et al*., [22] 1 case.

## Discussion

In the present case, eradication of HP infection obtained with a short course of antibiotic therapy resulted in a complete pathologic remission of a diffuse large B cell lymphoma of the stomach. This complete regression of the disease was confirmed not only by gastroscopy and biopsies but also by gastrectomy.

This finding confirms one more time that large B cell HP-positive gastric lymphomas are not necessarily associated with loss of HP dependence. Until few years ago, large B cell gastric lymphoma was considered independent of *Helicobacter pylori *stimulation. This assertion was supported by *in vitro *and *in vivo *results.

A study by Hussell *et al*., [4] showed that cells of a large B cell gastric lymphoma did not proliferate *in vitro *in response to *Helicobacter pylori*, as MALT lymphoma cells did.

*In vivo *confirmation came from the fact that a number of cases of antibiotic-resistant MALT lymphoma contained large B cells in deep layers of the stomach and these cells were thought responsible for absent response of these tumors [5,6]. Boot *et al*., [26] concluded that antimicrobial treatment should not be chosen as primary therapy for high grade MALT Non Hodgkin lymphoma, but additional *Helicobacter pylori *eradication could play a part in optimum treatment of an accompanying low grade component.

In 1997, Rudolph *et al*., [11] described a patient affected by DLBCL with areas of MALT lymphoma that responded to antimicrobial therapy. After few months, Seymour *et al*., [12] reported the case of a 73 year-old woman with a DLBCL and *Helicobacter pylori *associated chronic active gastritis; she refused chemotherapy and received only eradication treatment with an unexpected tumor remission. These two cases were the first published cases of regression of large B cell lymphoma after eradication therapy. Afterwards analogous surprising situations were reported.

Morgner *et al*., [15], collecting 8 cases of lymphoma regression, underscored the possible role of antimicrobial therapy in the treatment of gastric large B cell lymphoma. When this approach was studied as first line therapy for gastric large B cell lymphoma in clinical trial, encouraging results were obtained: there was a complete remission in 64% of cases (14 of 22) for Chen *et al*., [7], in 50% (2 of 4) for Hiyama *et al*., [16] and in 50% (5 of 10) for Nakamura *et al*., [10]. Alpen *et al*., [22] started a pilot-trial to investigate the role of HP eradication therapy in early gastric high-grade B-cell lymphoma prospectively. So far, two patients were treated, both patients become HP-negative after eradication therapy: one patient achieved CR. And the second patient received only a partial remission of the lymphoma. These studies present some limitations: as they include few patients; patients enrolled by Chen *et al*., [7] and Hiyama *et al*., [16] are a well defined subgroup characterized by clinical stage E I and presence of areas of MALT lymphoma; clinical stage is not clear in patients with high grade or low with focal high grade enrolled by Nakamura *et al*., [10]. Alpen *et al*., [22] in their study included patients with early high-grade gastric B-cell lymphoma at stage E I.

These authors paid attention to different prognostic factors. Hiyama *et al*., [16] focused on cytogenetic features, but they did not find any suggestive factor. Two largest trials indicated the depth of infiltration of tumor as the determinant factor for the complete remission: 100% (7 of 7) of tumors limited to mucosa or submucosa versus 30% (3 of 10) of those infiltrating to or beyond muscolaris propria achieved a complete remission as reported by Chen *et al*., [7]; for Nakamura *et al*., [10], 93% of all tumors (high and low grade) limited to the mucosa versus 23% of those demonstrating deep invasion of the submucosa or beyond obtained a complete remission.

In this review of the literature, age, sex, location of tumor and the presence or absence of areas of MALT lymphoma don't seem to influence the response of anti Helicobacter therapy. Clinical stage and depth of tumor invasion are the most important predictive factors of complete remission [27]. However it must be emphasized that locally-advanced stages can respond to the eradication treatment too. In some cases in stage beyond E I, there was a complete response of DLBCL in terms of gastric localization, but with persistent nodal disease[12,15]; surprisingly, in a patient, MALT lymphoma was detected after eradication treatment, while large B cell component was disappeared[15].

Very little is reported about genetics of these tumors [28,29]. According to the lymphoma MALT concept proposed by Isaacson and Wright [30], there is a sequence of events without solution of continuity from acquisition of gastric MALT, in most cases because of a *Helicobacter pylori *infection, to MALT lymphoma and large B cell lymphoma. There is consistent evidence for the clonal link between the small cell tumor and the large cell tumor [31]. This evolution is possible in t(11;18)(q21;q21) negative MALT lymphoma after the accumulation of some genetic aberrations which progressively increase its genetic instability [32]. t(11;18)(q21;q21) positive lymphoma does not transform itself and it does not accumulate genetic anomalies, but it has an aggressive course and is resistant to Helicobacter pylori eradication [33]. Therefore, two groups of DLBCL can be identified: one derives from a t(11;18)(q21;q21) negative MALT lymphoma; one, which contains less numerical genetic aberrations, arises *de novo *[32]. Not all DLBCLs without areas of MALT lymphoma arise *de novo*. The absence of the low grade component could be due to sampling bias or to overgrowth by large cells [31]. It is unknown if DLBCLs regressed after *Helicobacter pylori *eradication have a common genetic pattern and if cases without areas of MALT lymphoma are transformed lymphomas or *de novo *lymphomas.

The gold standard of treatment of primary gastric DLBCL is still controversial. The treatment of localized (stage EI and EII) disease was based on surgery alone, or followed by chemotherapy and/or radiotherapy, however recent studies showed that clinical outcome of localized gastric lymphoma treated by systemic chemotherapy alone was similar to that treated by surgery followed by chemotherapy in terms of tumor response, disease-free survival and overall survival suggesting that surgery be reserved for those with residual lymphoma after chemotherapy [34-38].

According to this review, among patients with complete remission obtained after eradication therapy, only one patient, who was affected by HIV infection, relapsed. These data suggest that after a complete remission, no other treatment including gastrectomy might be necessary, even if full thickness of gastric wall is infiltrated at presentation.

In most cases of gastric MALT lymphoma remission is achieved within 12 months after *Helicobacter pylori *eradication, but a late response of up to 45 months has been described [39]. Among these 42 cases of primary gastric large B cell lymphoma that obtained a complete remission after eradication treatment, the median time from the end of the therapy to the demonstration of remission was 8 weeks. If there were not signs of initial or complete response at the first endoscopic control (4–6 weeks after the end of eradication treatment), it was a contraindication to continue follow-up and an indication to conventional treatment [7,10,22]. Alpen *et al*., [22] submitted patients with only a partial response to chemotherapy/radiotherapy two months after the end of eradication therapy. Hiyama *et al*., [16] extended the follow-up to six months from the end of eradication therapy, at that point patients with partial or no response were treated with chemotherapy.

In all cases that responded to eradication therapy, initial or complete regression of lymphoma was evident at the first endoscopic and histologic examination. Only in one case, there was a disease progression, after an initial response, at the second examination [22].

## Conclusion

Our case reported here and the review of the literature allow us to conclude that:

1. Complete remission was obtained after HP eradication treatment in 42 of 61 patients with primary gastric HP related DLBCL.

2. There is no marker that can predict if the tumor will regress after antimicrobial therapy. However, depth of gastric wall infiltration and clinical stage can strongly predict the probability of a complete remission, it must be emphasized that complete remission was reached in anecdotal cases independently of these factors after anti HP eradication.

From a practical point of view we suggest that all patients with primary gastric DLBCL associated with *Helicobacter pylori *infection a complete staging with endoscopic ultrasonography, computed tomographic imaging and bone marrow biopsy should be carried out and the patients should first be treated by anti HP treatment. An endoscopic revaluation 4–6 weeks after the eradication treatment should be performed. These evolutions of lymphoma can happen:

- No response or progression: patient must undergo to surgery or other conventional treatment.

- Partial remission: lymphoma is probably responsive and could obtain a complete remission; patient must be strictly monitored to detect signs of progression or a complete remission.

- Complete remission: patient must be strictly monitored but may not require further treatments.

## Competing interests

The author(s) declare that they have no competing interests.

## Authors' contributions

LC diagnosed and treated the patient, revised and finally approved the manuscript for been published, RP performed bibliographic research and participated in manuscript revision process, PS performed bibliographic research and participated in manuscript revision process, AZ and CP performed pathological diagnosis and histological pictures. All authors read and approved the final manuscript.
